# Enabling the complete valorization of hybrid *Pennisetum*: Directly using alkaline black liquor for preparing UV-shielding biodegradable films

**DOI:** 10.3389/fbioe.2022.1027511

**Published:** 2022-12-05

**Authors:** Haojiang Qian, Yafeng Fan, Jiazhao Chen, Linsong He, Yongming Sun, Lianhua Li

**Affiliations:** ^1^ Guangzhou Institute of Energy Conversion, Chinese Academy of Sciences, Guangzhou, China; ^2^ Nano Science and Technology Institute, University of Science and Technology of China, Suzhou, China; ^3^ Guangzhou Institute of Energy Conversion, CAS Key Laboratory of Renewable Energy, Chinese Academy of Sciences, Guangzhou, China; ^4^ Guangdong Key Laboratory of New and Renewable Energy Research and Development, Guangzhou, China

**Keywords:** energy grass, hybrid *Pennisetum*, alkaline black liquor, film, UV-shielding, lignin, biorefinery

## Abstract

The conversion of lignocellulosic biomass into various high-value chemicals has been a rapid expanding research topic in industry and agriculture. Among them, alkaline removal and utilization of lignin are important for the accelerated degradation of biomass. Modern biorefinery has been focusing the vision on the advancement of economical, green, and environmentally friendly processes. Therefore, it is indispensable to develop cost-effective and simple biomass conversion technologies to obtain high-value products. In this study, the black liquor (BL) obtained from the alkaline pretreatment of biomass was added to polyvinyl alcohol (PVA) solution and used to prepare degradable ultraviolet (UV) shielding films, achieving direct and efficient utilization of the aqueous phase from alkaline pretreatment. This method avoids the extraction step of lignin fraction from black liquor, which can be directly utilized as the raw materials of films preparation. In addition, the direct use of alkaline BL results in films with similar UV-shielding properties, higher physical strength, and similar thermal stability compared with films made by commercial alkaline lignin. Therefore, this strategy is proposed for alkaline-pretreated biorefineries as a simple way to convert waste BL into valuable products and partially recover unconsumed sodium hydroxide to achieve as much integration of biomass and near zero-waste biorefineries as possible.

## Highlights

• Prepare anti-ultraviolet film by directly using alkaline pretreatment black liquor.
• Adding black liquor to polyvinyl alcohol enhanced the performance of composite film.
• Black liquor alkaline pretreatment can be directly utilized to the maximum extent.
• Combine with current technology, zero waste liquid biorefinery can be realized.

## 1 Introduction

Lignocellulosic biomass is widely considered as a sustainable source of biofuels and biochemicals (Usmani et al., 2021; Kumar et al., 2022; Saravanan et al., 2022) *via* various techniques such as thermochemical, biological, and mechanical treatments (Periyasamy et al., 2022; Sidana and Yadav, 2022; Zhou and Tian, 2022). In the area of biological valorization, most efforts are focused on valorizing the saccharides (mainly hemicellulose and cellulose) into targeted products such as biogas, bioethanol, and other valuable chemicals (Ghimire et al., 2021; Devi et al., 2022; Raj et al., 2022), while the presence of lignin significantly suppresses the biodegradation activities and needs to be removed (Areepak et al., 2022; Mikulski and Klosowski, 2022; Nordin et al., 2022). Alkaline pretreatment is effective for biomass delignification, and normally, ideal lignin removal performance can be achieved when grass biomass is alkaline pretreated under mild conditions. Recent studies on alkaline pretreatment on hybrid *Pennisetum* achieved 68.6% lignin removal at 35°C for 24 h (Kang et al., 2018), and the obtained cellulose-rich residue was used for bioethanol and biogas production *via* singular and co-production scenarios. It achieves conversion of biomass for biofuel production, but the aqueous phase after pretreatment (mainly including lignin and a fraction of hemicellulose) still remains unused components and is considered waste. The conversion of alkaline lignin to various products (e.g., biochar and biochemicals) has been extensively studied (Jin et al., 2022; Kim et al., 2022; Paul et al., 2022; Xiu et al., 2022), whereas the direct utilization of lignin-rich alkaline black liquor (BL) for valuable products has been seldomly reported.

Recently, the usage of lignin as an additive for preparing polyvinyl alcohol (PVA)-based films has emerged as a method of using lignin in the material context. By simply adding lignin to PVA gels, the obtained films can exhibit higher mechanical strength and UV-shielding properties (Posoknistakul et al., 2020; Zhang et al., 2020; Ma et al., 2022). However, in identical practice, commercial lignin needs to be obtained as another co-ingradient. Obtaining lignin from alkaline BL requires a cost-addition process for lignin extraction and purification. Thus, if alkaline BL can be straightly and sufficiently used as an additive for PVA films, the process cost can be reduced. In addition, the successful direct utilization of alkaline BL as a valuable raw material for UV-shielding films will enable the development of integrated techniques for near-complete valorization of lignocellulosic biomass. However, no studies have reported the direct usage of BL as an additive for biodegradable plastic materials.

Thus, this study was conducted to (1) directly use alkaline BL as an additive for preparing UV-shielding biodegradable films, (2) characterize and analyze the properties of the obtained PVA/BL films compared with those of PVA/alkaline lignin (PVA/AL) films, and (3) analyze the composite film formation mechanisms. In addition, a brief discussion has been provided on the feasibility of the co-production of UV-shielding films and biofuels for the complete valorization of lignocellulosic biomass.

## 2 Materials and methods

### 2.1 Materials

Hybrid *Pennisetum* was collected from the Zengcheng District, Guangzhou, China, in November 2021. To collect 60–100 mesh particles for further use, the raw biomass was dried, ground and sieved. The total solid (TS) and volatile solid (VS.) contents were 96.71 ± 0.06 and 85.30 ± 0.14%, respectively. The cellulose, hemicellulose, and lignin contents of the samples were 35.33 ± 2.74, 19.08 ± 2.58, and 18.61 ± 0.47%, respectively. Sodium hydroxide (NaOH, 95%) and polyvinyl alcohol (1799-PVA) were purchased from Shanghai McLean Biochemical Co., Ltd. Glucose, xylose, and arabinose standards were obtained from Aladdin Biochemical Technology Co. Ltd. (Shanghai, China). Commercial alkaline lignin was purchased from Shanhu Chemical Co. Ltd. (Nanjing, China). All the chemicals were used without further purification.

### 2.2 Preparation of black liquor and polyvinyl alcohol glue

The alkaline BL was the liquid fraction collected from alkaline pretreatment on hybrid *Pennisetum* with 6 wt% NaOH aqueous solution at 37°C for 24 h, as described in a previous study (Kang et al., 2018) from our group, which is the optimized condition for alkaline pretreatment on Hybrid *Pennisetum*. The obtained BL was used directly without addtional treatment. PVA glue (10 wt%) was prepared using a method introduced in recent studies (Zhang et al., 2020; Yang et al., 2021).

### 2.3 Preparation of the composite films

The preparation of PVA/BL and PVA/AL films was an upgraded method based on previous studies (Xiong et al., 2018; Huang et al., 2021; Yang et al., 2021). First, 50 g of PVA glue (10 wt%) was added to a conical flask (volume 100 ml). Next, alkaline lignin/NaOH or alkaline BL was added to the PVA glue, as detailed in Tables 1, 2, respectively. Different volumes of the solution were selected to achieve a composition of lignin in the film from 0 to 10%. Subsequently, the mixtures were stirred constantly (250 rpm) for 3 h at 60°C, followed by cooling to room temperature (25 ± 10°C). Finally, each film required 2 g of mixed glue solution to be added to a polytetrafluoroethylene (PTFE) column mold (Φ = 5 cm) and evaporated at 35°C for 48 h to form the films, which were then rinsed with deionized water and dried. The samples were labeled according to the type and concentration of additives. It should be noted that the percentages on the labels represent the mass ratio of the lignin content in the BL/AL additives and PVA.

**TABLE 1:** Preparation parameters of PVA/BL films.

Sample	Proportion (wt%)	Lignin mass (g)	Black liquor (ml)
PVA-0.5%BL	0.5	0.025	1.65
PVA-1.0%BL	1.0	0.050	3.29
PVA-3.0%BL	3.0	0.150	9.88
PVA-5.0%BL	5.0	0.250	16.47
PVA-10.0%BL	10.0	0.500	32.93

**TABLE 2:** Preparation parameters of PVA/AL films.

Sample	Proportion (wt%)	Alkali lignin (g)	NaOH solution (ml)
PVA-0.5%AL	0.5	0.025	1.65
PVA-1.0%AL	1.0	0.050	3.29
PVA-3.0%AL	3.0	0.150	9.88
PVA-5.0%AL	5.0	0.250	16.47
PVA-10.0%AL	10.0	0.500	32.93

### 2.4 Properties of composite films

#### 2.4.1 Ultraviolet and visual light absorption

The UV absorption properties of the films were measured using a UV-Visible-NIR-Spectrophotometer (Lambda PerkinElmer, United States). Each film was cut into squares of 3 cm × 3 cm and fixed to a mold for analysis. Each sample was scanned at 260 nm/min within the range of 200 nm–760 nm. Each test was repeated six times.

#### 2.4.2 Mechanical strength

The mechanical properties of the films were tested using a universal mechanical testing machine (INSTRON 5982) according to the ISO 179-1993 standard. In each test, the dumbbell-shaped specimen (7.50 cm × 5.00 mm) was mechanically stretched, a load cell sensor of 200–250 N was used, the crosshead moving speed was 5 mm/min, the initial fixture spacing was 4.00 cm, and the experiment was conducted at 25°C and relative humidity of 46%. A minimum of five tensile tests were conducted for each sample.

#### 2.4.3 Water uptake and swelling properties

The water absorption and swelling properties of the films were tested by soaking them in deionized water for 48 h at room temperature. After soaking, the samples were wiped with filter paper (Φ = 90 mm) to remove surface water. The weight and thickness before and after soaking were measured, and the equilibrium swelling rate (ESR) and water retention (WR) rate were calculated as per Eqs 1, 2, respectively. Each test was performed in triplicate, and the average value is presented as the relative standard deviation as the error bar.

WR=Ws / Wd (1)

ESR=(Ws−Wd)/ Wd (2)

Where *W*_*d*_ represents the dry weight and *W*_s_ represents the weight after swelling (Pan et al., 2006; Tong et al., 2007; Wu et al., 2019).

#### 2.4.4 Surface roughness

The surface roughness of the films was characterized using atomic force microscopy (AFM; Multimode 8 Bruker, United States). For each sample, a square area of 5 μm × 5 μm was scanned at a rate of 0.996 Hz and consisted of 256 lines. The probe model used was RFESPA-75 (f_0_ = 75 kHz, k = 3 N/m, Bruker, United States). Air-soft tapping was applied as the test mode, and the data were analyzed using NanoScope Analysis 1.5. The roughness of the composite film is specified by the magnitude of the values of Rq and Ra, where Rq represents the root mean square value and Ra represents the average value of the relative datum plane.

#### 2.4.5 Thermal stability

The thermal stability of the films was tested by thermogravimetric analysis (TGA; SDT650 TA, United States) in the range of 25–700°C at a heating rate of 10°C/min, with nitrogen (feeding rate: 40 ml/min) as the protection gas.

#### 2.4.6 Differential scanning calorimetry

The thermophysical properties of materials were analyzed by low differential scanning calorimetry (DSC6000, PerkiElmer, Netherlands). The samples were heated from 25°C to 230°C at 10°C/min under argon for 3 min to remove the volatile components and thermal history. Then the samples were cooled to 25°C at the same rate and heated to 230°C after 3 min, during which the glass transition temperature of (Tg) the samples were recorded.

### 2.5 Functional groups of film materials

The surface functional groups of the samples were analyzed using a Fourier transform infrared spectrometer (FTIR, Nicolet Is 50, Thermo Fisher Scientific) in the attenuated total reflection mode (IS5O ATR). Each scan was conducted in the range of 4,000–800 cm^−1^ with automatic gain on.

### 2.6 NMR characterisation of lignin

The black liquor was adjusted to acidity, the solids were precipitated and extracted, and two-dimensional heteronuclear single-quantum coherent NMR spectroscopy (2D-HSQC-NMR) was performed on lignin samples using a Bruker AVANCE III 400 MHz NMR spectrometer equipped with a PABBO probe. A 50 mg solid sample was dissolved in 0.6 ml of DMSO-d_6_ and HSQC spectroscopy was performed at a relaxation delay of 2 s. The percentages of G, H and S units and the S/G ratio were according to the calculations presented in a previous study (Wen et al., 2013).

### 2.7 Concentration of NaOH in rinsed water

The concentration of NaOH in the solution collected from the rinsing films was quantified by testing the alkalinity using an automatic potentiometric titrator (APT; YLB-Titron Line Easy, Julabo, Germany). In this test, the endpoint pH was set to 7, and the endpoint determination was delayed for 1 s. The standard curve is provided in the supplementary material. Alkalinities of various NaOH/water mixtures is shown in Supplementary Figure S1 in the supporting materials.

## 3 Results and discussion

### 3.1 Properties of polyvinyl alcohol films with the addition of alkaline lignin and alkaline black liquor

As mentioned above, the exact properties of PVA/BL films remain unknown. In this study, the diverse properties of PVA/BL films based on multiple characterizations were analyzed and compared based on the properties of PVA/BL and PVA/AL.

#### 3.1.1 Light transmission properties of various composite films

**FIGURE 1:**
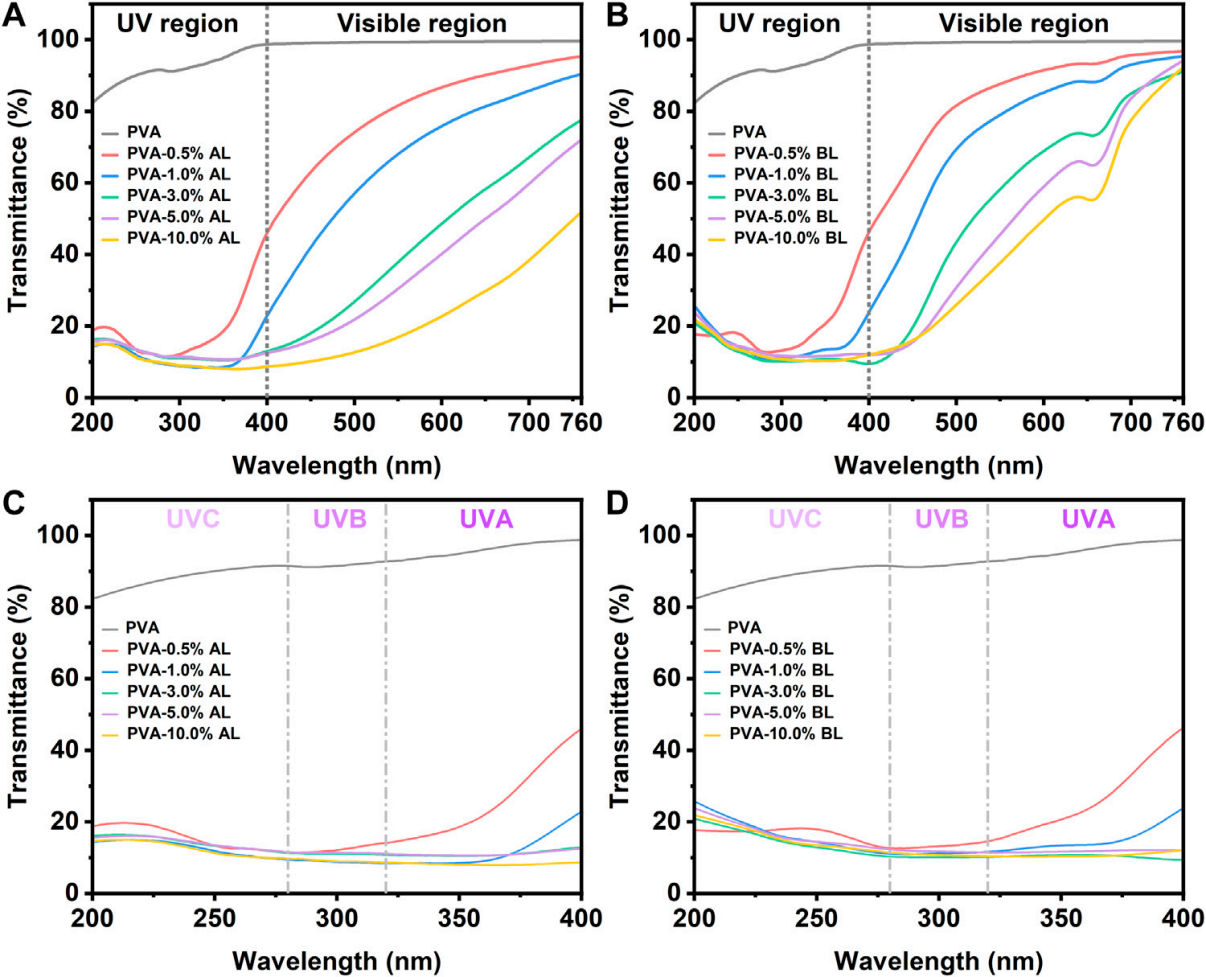
Light transmittance of PVA, PVA/AL, and PVA/BL films. **(A)** 200–760 nm AL; **(B)** 200–760 nm BL; **(C)** 200–400 nm AL; **(D)** 200–400 nm BL.

Figures 1A,B show the full-band (wavelength at 200–760 nm) light transmittance of the PVA/AL and PVA/BL films, respectively. The PVA films had light transmittances of 81–100% in the UV region (200–400 nm) and 100% in the visible region (400–760 nm), which is compatible with previous studies on the optical properties of PVA (Wang Y. et al., 2017b; Huang et al., 2021; Van Nguyen and Lee, 2022). Notably, with the addition of only 0.5% AL, the transmittance of UV light in the UV region significantly declined, which range from 12 to 42%. The amount of AL was further increased to more than 1.0% and the light transmittance was less than 25%. Therefore, the use of lignin as an additive can significantly enhance the UV-shielding performance of PVA films.

However, the addition of AL also resulted in the absorption of visible light. Figure 1A reveals that the transmittance of visible light gradually decreased with increasing AL. Specifically, the transmittance of visible light for the PVA/AL film with 0.5% AL ranged from 45 to 90%, whereas when 10.0% AL was added, it reached 10–50%. Interestingly, adding BL directly into PVA achieved similar UV-shielding performance; meanwhile, the transmittance of visible light also declined with increasing BL content, as shown in Figures 1C,D. Interestingly all the PVA/BL films observed slight fluctuations at wavelengths near 660 nm, a particular phenomenon that deserves further investigation.

Collectively, the appropriate amount of alkaline pretreatment-derived black liquor can be used directly as an additive to improve the optical properties of PVA-derived biodegradable films, whose high transparency and strong UV absorption properties are of great value for packaging materials and have many potential applications (Akhramez et al., 2022; Liu et al., 2022; Zhang et al., 2022).

#### 3.1.2 Mechanical properties of various composite films

**FIGURE 2:**
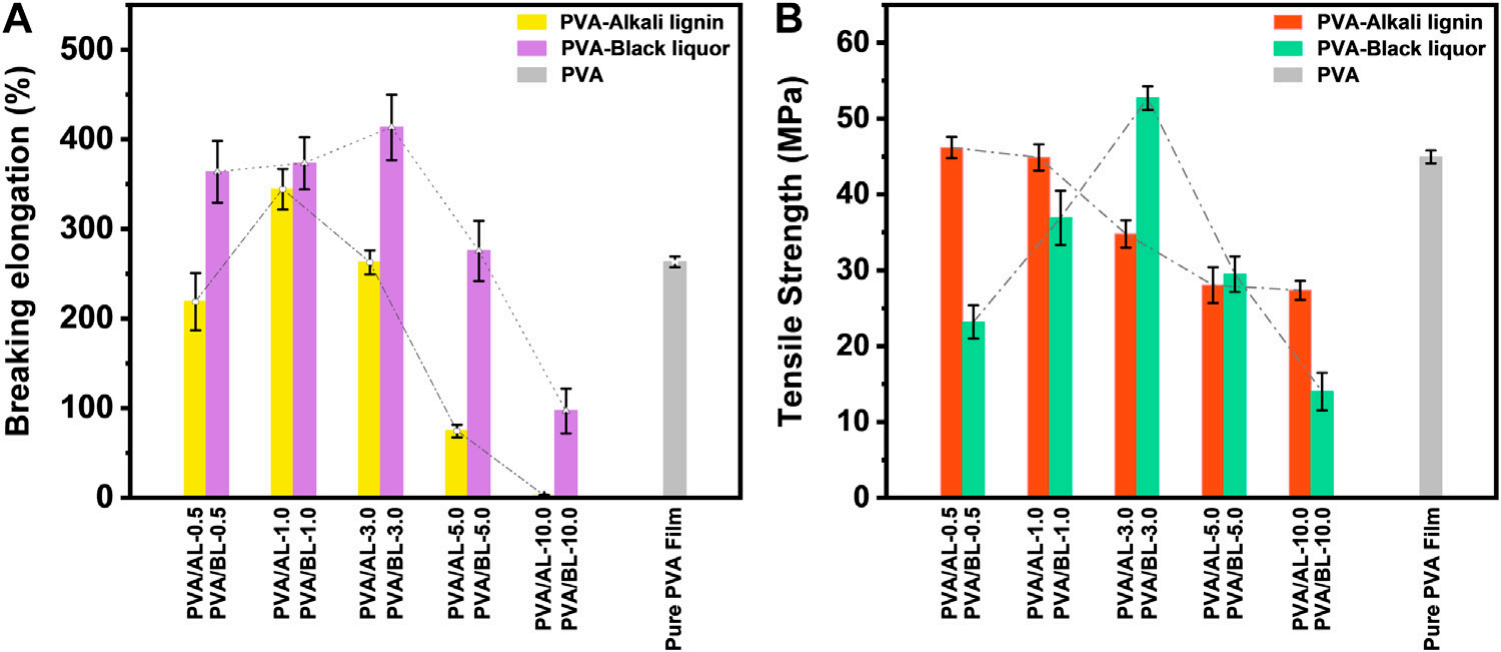
Mechanical Properties of various films. **(A)** breaking elongation of various composite films; **(B)** tensile strength of various composite films.

The breaking elongation and tensile strength are critical indices for film materials. The former represents the soft and elastic properties of the materials, whereas the latter represents the resistance of the materials to the material to the maximum uniform plastic deformation. Figure 2A illustrates the breaking elongations of the films. The elongation of the PVA film was 263.32%. As for the breaking elongation of the two kinds of composite films, both showed a similar tendency with the addition of AL and BL. Specifically, the breaking elongation displayed an obvious increase with the addition of AL and BL; afterwards, it reached a peak of 344.28% and 413.32%, where the addition of AL and BL were 1.0% and 3.0%, respectively. When successive additions of AL and BL were made, there was an obvious decrease, but the elongation at break of the PVA/AL film was relatively low, which could be attributed to the enhanced intermolecular forces of the polymer due to the hydrolysis products of cellulose and hemicellulose in the black liquid (Gao et al., 2014). Figure 2B shows the tensile strengths of the films. It is clear that the maximum value of PVA/BL (52.70 Mpa) is higher than that of PVA/AL (46.19 Mpa) with the same variation in AL and BL addition, which in combination with the relevant literature can be explained by the high efficiency of hydrogen bonds entangled between various compounds such as lignin, hemicellulose derivatives and PVA in the black liquor. In addition, the variation in tensile strength of the composite film in the range 0.5–10.0% BL may be due to the dispersion state of the additive in the matrix (Guan et al., 2014; Xiong et al., 2018). In view of the higher breaking elongation and tensile strength of PVA/BL films, it is evident that PVA/BL showed superior mechanical durability with a BL of 3.0% compared with other films.

#### 3.1.3 Surface roughness of various composite films

**FIGURE 3:**
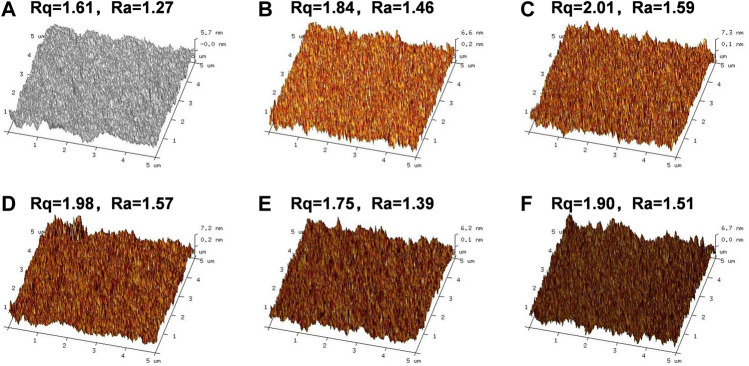
Atomic Force Microscope (AFM) of PVA and PVA/AL films. **(A)** PVA; **(B)** PVA-0.5%AL; **(C)** PVA-1.0%AL; **(D)** PVA-3.0%AL; **(E)** PVA-5.0%AL; **(F)** PVA-10.0%AL.

**FIGURE 4:**
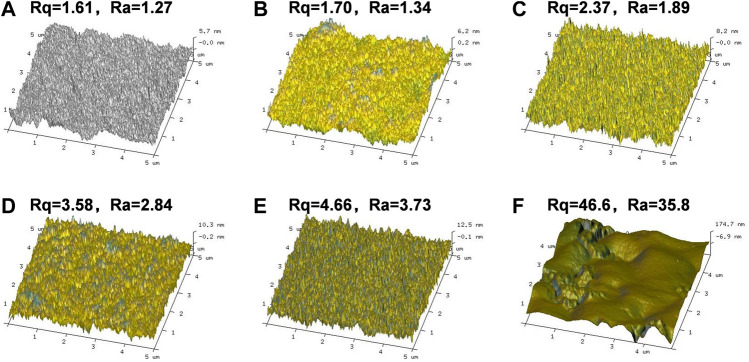
Atomic Force Microscope (AFM) of PVA and PVA/BL films. **(A)** PVA; **(B)** PVA-0.5%BL; **(C)** PVA-1.0%BL; **(D)** PVA-3.0%BL; **(E)** PVA-5.0%BL; **(F)** PVA-10.0%BL.

Figures 3, 4 present the AFM images of various films. It is plausible to conclude that adding AL or BL to PVA caused a slight increase in the surface roughness of the obtained films. Specifically, when the BL content reached 10.0%, the root means square value (Rq) and average value of the relative datum plate (Ra) increased to 46.6 and 35.8, respectively. Compared with the PVA/AL films, both the Rq and Ra of PVA/BL were substantially higher, indicating that using BL as the additive drastically increased the surface roughness.

#### 3.1.4 Characterization of composite films based on other basic properties

Data for the PVA films were not obtainable because PVA dissolved in deionized water during soaking (Supplementary Figure S2) which presents the water absorption and swelling ratios of the films. Both the PVA/AL and PVA/BL films showed a similar trend in ESR and WR, with an increasing trend from 0.5 to 3.0%, followed by a decrease. Thus, the PVA/BL films exhibited water uptake and swelling properties similar to those of the PVA/AL films.

**FIGURE 5:**
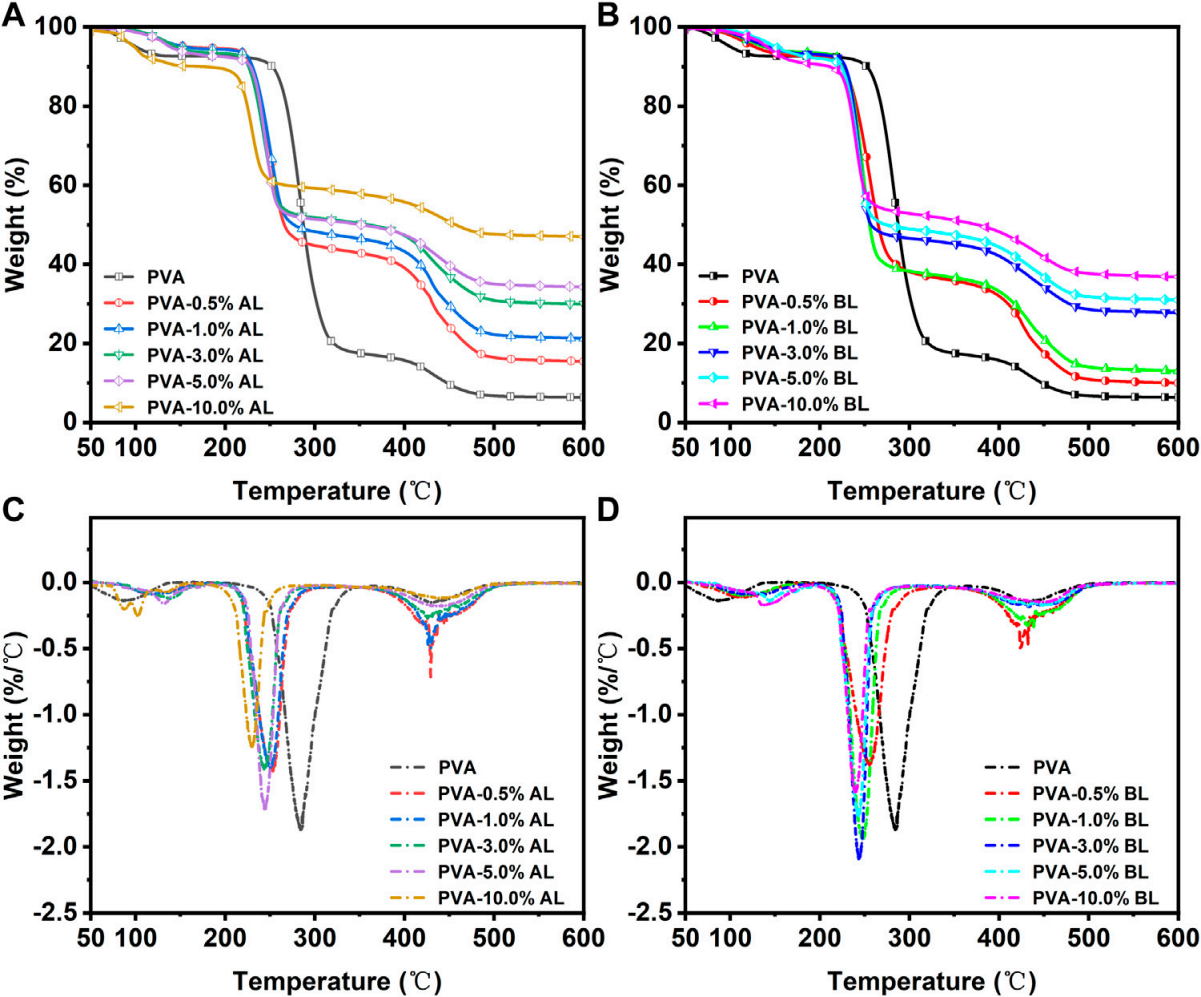
Thermal Stability of various composite films. **(A,C)** present the TG and DTG spectra of PVA and PVA/AL films; **(B,D)** present the TG and DTG spectra of PVA and PVA/BL films.

Figures 5A,C show the weight loss (%) and weight-loss rates (%/°C) of the PVA and PVA/AL films at 50–600°C. The weight loss of the PVA film can be divided into three stages: (Stage 1) water evaporation (50–140°C), (Stage 2) decomposition and degradation of PVA (234–350°C), and (Stage 3) chain scission and cyclization reactions (395–500°C). A similar description has been provided in previous studies and has not been discussed further (Zhao et al., 1998; Alexy et al., 2001; Dong et al., 2014; Dong et al., 2016). The addition of AL caused differences in the thermostability of Stages 2 and 3. Specifically, the decomposition of compounds advanced to 200°C, which is related to the decomposition/evaporation of some light compounds (e.g., lignin monomers and oligomers). Less weight loss was achieved, likely due to interactions such as the hydrogen bond crosslinking between lignin and PVA, which formed compounds that were difficult to decompose. Further, the addition of AL resulted in less weight loss at Stage 3 and gradually decreased with the increase in AL content. As shown in Figures 4B,D, the addition of BL suppressed the thermal decomposition of the obtained films and exhibited a trend similar to that of the PVA/AL films. However, the addition of BL resulted in slightly more weight loss at both Stages 2 and 3 compared with the addition of AL. This is likely due to the decomposition of carbohydrates in the BL as carbohydrates have lower pyrolytic temperatures than lignin (Sousa et al., 2016; Rao et al., 2021).

**FIGURE 6:**
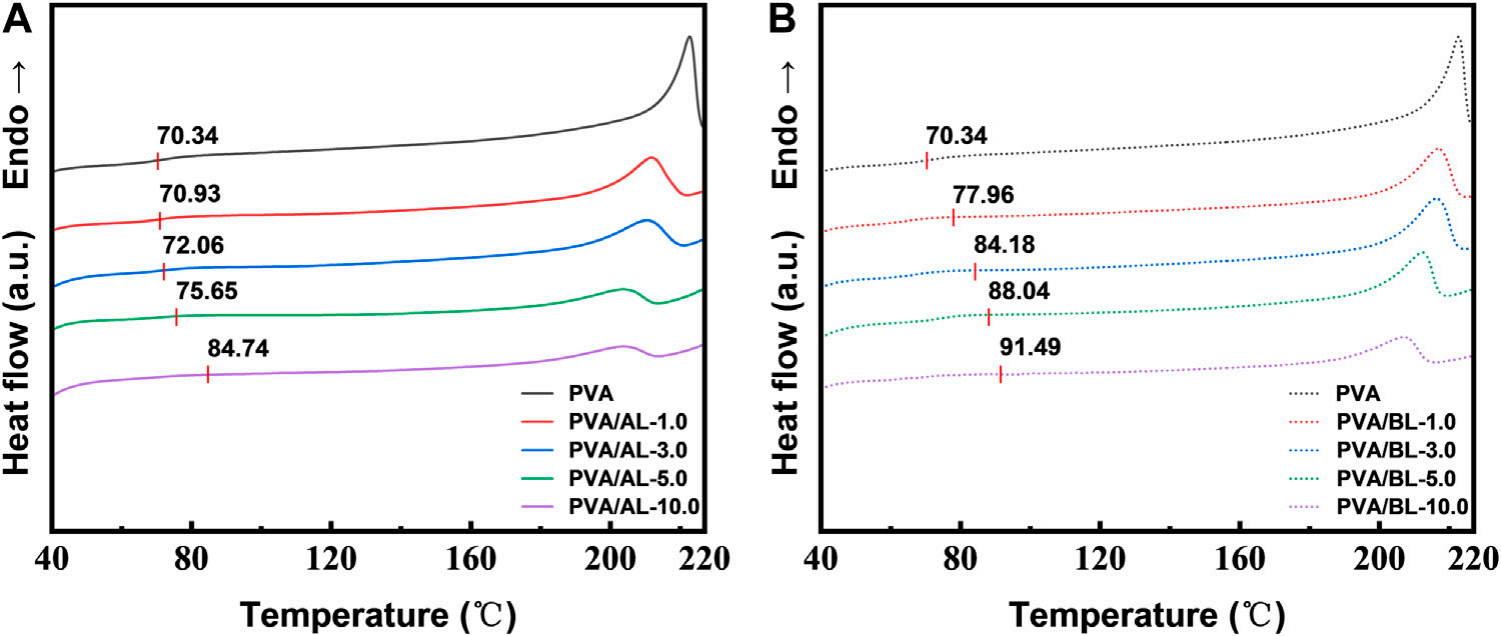
Differential scanning calorimetry (DSC) of various composite films. **(A)** PVA-AL; **(B)** PVA-BL.

Figures 6A,B show the warming transition of two polymeric materials respectively. It was observed that the glass transition temperatures (Tg) of the composite films were both higher than those of the pure PVA films (70.23°C). The values for Tg increased from 70.93 to 83.5°C when increasing the AL content from 1.0 to 10.0%. This should be attributed to the hydrogen bonding between the alkali lignin and PVA molecules, limiting the degree of free movement of the PVA molecular chains, which is consistent with relevant lignin-based materials reported in the literature (Xiong et al., 2018; Zhang et al., 2020). As the BL content increased from 1.0 to 10.0%, the Tg value increased from 77.96 to 88.60°C. In contrast, the glass transition temperature of the black liquor composite films showed a similar shift and had a higher glass transition temperature (Tg), because both the carbohydrate derivatives and lignin in the black liquor cross-linked with the PVA, increasing the rigidity of the molecular chains, and reducing the intermolecular mobility, thus increasing the glass transition temperature (Tg) of the polymer.

### 3.2 Discussion on the formation mechanisms of composite films

**FIGURE 7:**
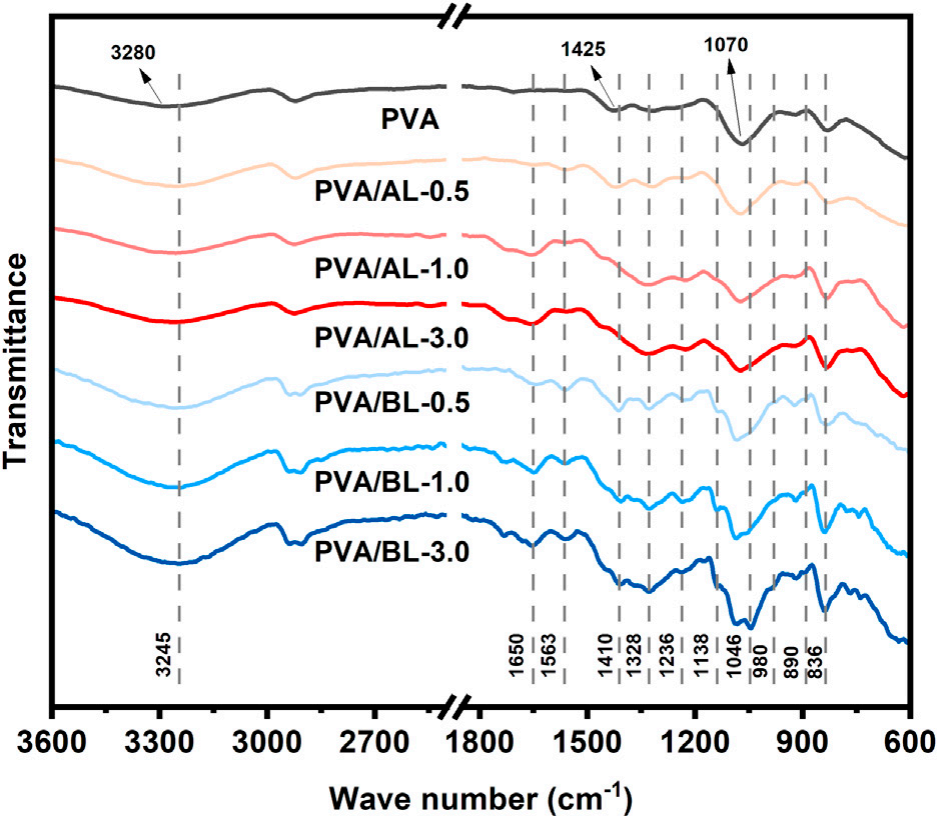
FT-IR spectra of PVA, PVA/AL, and PVA/BL composites.

As shown in Figure 7 and Supplementary Table S1, the FT-IR spectra of various films exhibited five main peaks at 3,280, 2,915, 1,425, 1,070, and 836 cm^−1^, corresponding to the intermolecular interaction of the alcohol hydroxyl group, CH_2_ group asymmetric stretching bands from the methyl group, deformation of O–H and C–H bonds in the plane, C–O bond out-of-plane vibration, and the appearance of the PVA skeleton band, respectively (Mahmud et al., 2006; Abdelrazek et al., 2010; Franca et al., 2022). Several changes were observed with the addition of alkaline lignin. The bands at 1,650, 1,563, 1,328, and 836 cm^−1^ are typical lignin characteristic peaks (Serrano et al., 2010; Wu et al., 2016; Jin et al., 2022). Further, the peak positions of some of the functional groups changed. The association vibration of the C–H bonds on the lignin S and H units and the CH_2_ groups of PVA occurred at 836 cm^−1^. In addition, the hydroxyl peak at 3,280 cm^−1^ shifted to 3,264 cm^−1^, and a similar shift from 1,070 cm^−1^ to 1,078 cm^−1^ was observed, which confirmed the strong crosslinking phenomenon between alkaline lignin and PVA, as found in previous studies (Kubo and Kadla, 2003; Huang et al., 2021).

With the addition of BL, additional peaks were observed. The bands at 1,650, 1,563, 1,328, and 836 cm^−1^ were assigned to lignin, whereas those at 1,236, 1,138, 1,046, 980, and 890 cm^−1^ were assigned to carbohydrates (e.g, glucan, xylan) (Peng et al., 2014; Sun et al., 2014; Wang F. L. et al., 2017a; Liu X. W. et al., 2019a; Boukir et al., 2019). The addition of BL also caused changes in the characteristic PVA peaks. Compared with the PVA film, there was a red shift at 3,280 cm^−1^. Unlike the blend of alkaline lignin, the BL composite films had a more obvious shift from 3,280 cm^−1^ to 3,245 cm^−1^ and showed a strong broad peak, combined with the characterization results of DSC and previous reports, which confirmed that hydrogen bonding between carbohydrates in BL and PVA formed (Liu X. X. et al., 2019b). Additionally, there is a double peak absorption in the range of 3,000–2,880 cm^−1^, which is attributed to the mixture of methyl, methylene, and asymmetric stretching vibrations owing to the complicated compositions of BL (Posoknistakul et al., 2020; Zhang et al., 2020).

### 3.3 HSQC NMR spectra of lignin in samples

**FIGURE 8:**
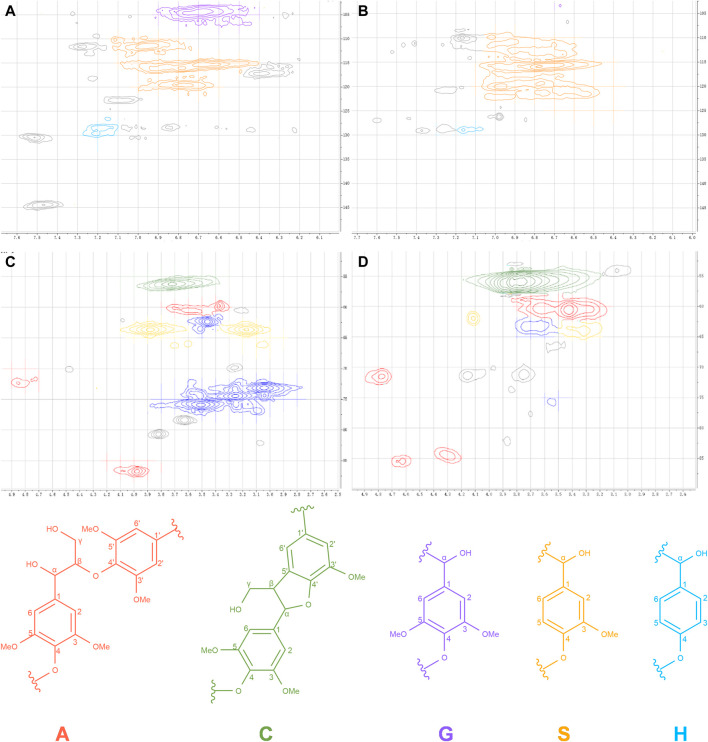
2D-NMR spectra for lignin. **(A,C)** represent the aliphatic-oxygenated ^1^H−^13^C correlations (δH/δC 2.5–6.0/50–90 ppm region) giving information on lignin side-chain interunit linkages; **(C,D)** represent the aromatic/olefinic ^1^H−^13^C correlations (δH/δC 6.0–8.0/100–150 ppm region) giving information on lignin aromatic units.

For the lignin-rich black liquor fraction, lignin was collected, characterized with 2D-NMR, and compared with alkaline lignin. NMR spectra for the δ_H_/δ_C_ 2.6–6.0/50–90 and 6.0–8.0/100–150 (aromatic and fatty) ppm regions are given in Figure 8, and the assignment of the main signals is shown in Supplementary Table S2 in the Supporting Information (Fan et al., 2021; Huang et al., 2021).

At anomeric regions (Figures 8A,B), significant differences were observed at δ_H_/δ_C_ 3.04–3.51/62.3–75.90 ppm. In brief, the correlation of δ_H_/δ_C_ 3.3–3.6/59–64 ppm was mainly manifested by the signal of C_γ_-H_γ_ in β-O-4′ substructure (A) overlapped with the signal of C_5_-H_5_ in β-D-xylopyranoside (X) and the signal of C_γ_-H_γ_ in phenylcoumaran substructures (C). The δ_H_/δ_C_ 73.23/3.04, 74.50/3.26, 75.90/3.51 ppm were attributed to the C_2_-H_2_, C_3_-H_3_, and C_4_-H_4_ units of β-D-xylopyranoside (X), respectively, which coincided with the FTIR detection of xylose-related outgoing peaks. No obvious signals were found for the X_2_, X_3_, X_4_, and X_5_ units in alkaline lignin, indicating that the release of xylan and the removal of lignin occurred simultaneously during the pretreatment and that some of the structures were connected.

The aromatic region corresponds to the vibration of the aromatic ring skeleton detected by FTIR and lignin fractions of both samples were quantified according to the results. As presented in Table 3, the pretreatment produced a higher content of lignin eugenol (S), which was significantly higher than the S/G of alkali lignin, further and indicating a difference in the distribution of lignin and alkali lignin in the black liquor, a result that may be one of the reasons for some of the performance differences between the two composite films.

**TABLE 3:** Aromatic unit structures of lignin.

Aromatic unit strutures	Lignin extracted from BL (%)	Akalin lignin (%)
S	44.97	2.96
G	48.28	87.18
H	6.75	9.86
S/G	0.931	0.034

### 3.3 Discussion on biomass valorization by integrated film synthesis and biofuel production

As mentioned above, using BL as the PVA additive is feasible for improving the UV-shielding performance of the obtained film and can achieve similar durability under various test conditions. Particularly, when 3.0% BL was added to the PVA glue, the PVA/BL films showed good ultraviolet absorption performance, mechanical strength, and thermostability, which were not worse or better than those of PVA/AL films. However, it must be noted that using BL directly caused higher roughness and could uptake more moisture content, which needs to be addressed in future studies. In view of these results, it is plausible to conclude that producing PVA/BL UV-shielding films directly is a suitable alternative means for valorizing alkaline BL.

**FIGURE 9:**
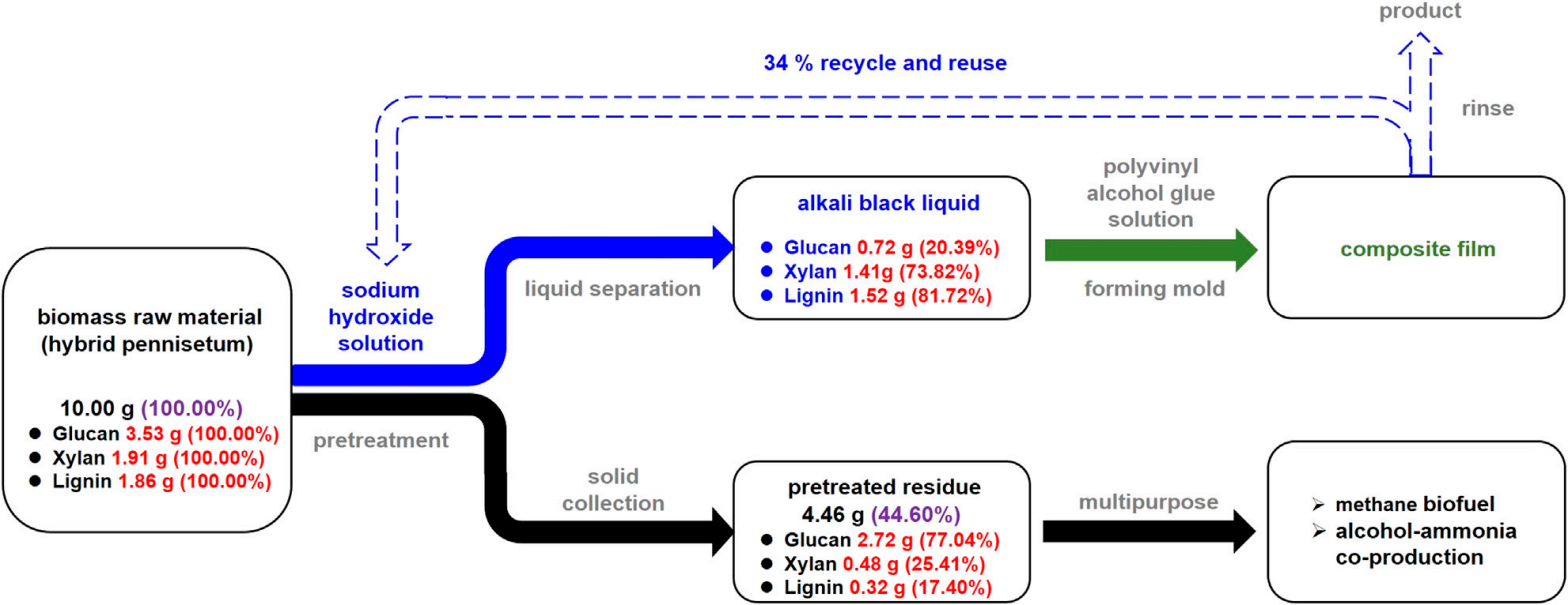
A proposed schematic of the integrated valorization of hybrid *Pennisetum* by co-producing biodegradable UV-shielding films and biofuels with mass flow.

Combined with the valorization of the delignified biomass, Figure 9 briefly shows the integrated production of UV-shielding biodegradable films and biofuels. In brief, alkaline pretreatment of 10 g biomass with NaOH (6 wt%) under 37°C for 24 h could produce a BL containing 1.52 g lignin, 0.72 g glucan, 1.41 g xylan, and some content of unconsumed NaOH. This BL can be added to a PVA solution, producing a UV-shielding film *via* the process proposed in this study. In addition, after film formation, the rinsing process could recycle 34% NaOH. The solid content, containing 2.72 g glucan, 0.48 g xylan, and 0.32 g lignin, was more suitable as a feedstock for its further application (e.g., producing biofuels *via* anaerobic digestion or fermentation). As investigated by Kang et al. (2018), alkaline- pretreated hybrid *Pennisetum* with 9.9% lignin is capable of producing methane at 257.6 ml/g VS In another study by Hosgun et al. (2017), this pretreated biomass achieved a glucose yield of 48.32%, which can be fermented for bioethanol production.

## 4 Conclusion

This study provides a simple and feasible approach for valorizing alkaline BL produced by biomass pretreatment. By mixing the BL with PVA solutions, the formed PVA/BL films exhibited excellent UV-shielding performance and physical durability. Specifically, adding 3.0% of alkaline BL achieved a film that suppresses the UV transmittance to less than 20%, with breaking elongation at higher than 400% and tensile strength higher than 50 MPa, which were better than the films prepared by commercial alkaline lignin. This simple approach can drastically reduce the cost of preparing PVA/lignin films by extracting lignin from biomass delignification waste streams. It is recommended as a means to achieve near-complete valorization of lignocellulosic biomass. In addition, by recycling the unconsumed NaOH *via* a simple rinsing process, lower pollutant emissions were achieved as far as possible. Further efforts are suggested to improve the properties of PVA/BL films, especially the surface smoothness and hydrophobicity.

## Data Availability

The original contributions presented in the study are included in the article/Supplementary Material; further inquiries can be directed to the corresponding author.
