# Supplementary material for: Enabling the complete valorization of hybrid Pennisetum: Directly using alkaline black liquor for preparing UV-shielding biodegradable films
Source: Front Bioeng Biotechnol. 2022 Dec 5;10:1027511. doi: 10.3389/fbioe.2022.1027511 (PMC9760701; doi:10.3389/fbioe.2022.1027511)
Supplement: Supplementary file 1 [file DataSheet1.docx]

**Enabling the complete valorization of hybrid *Pennisetum*: Directly using alkaline black liquor for preparing UV-shielding biodegradable films**

Haojiang Qian ^1, 2(†)^, Yafeng Fan ^1, 2^, Linsong He ^1^, JiaZhao Chen ^1^, Yongming Sun ^1, 2, 3, 4^, Lianhua Li ^1, 2, 3, 4,^ *

^1^ Guangzhou Institute of Energy Conversion, Chinese Academy of Sciences, Guangzhou 510640, P.R. China.

^2^ Nano Science and Technology Institute, University of Science and Technology of China, Suzhou 215123, P.R. China

^3^ Guangzhou Institute of Energy Conversion, CAS Key Laboratory of Renewable Energy, Chinese Academy of Sciences, Guangzhou 510640, P.R. China.

^4^ Guangdong Key Laboratory of New and Renewable Energy Research and Development, Guangzhou 510640, P.R. China.

***Corresponding authors**: Lianhua Li

Address: No. 2, Energy Road, Guangzhou, 510640, China

**Table S1.** Main bands assignments of FTIR in alkaline black liquor composite films.

| **Wavenumber (cm^-1^)** | **Band assignments** |
| --- | --- |
| 3600-3000  3000-2850  1650  1563  1410  1328  1236  1138  1046  980  890  836 | ѵ(OH) hydroxyl groups in lignin, dextran, xylan, partial monosaccharide, and polyvinyl alcohol (PVA) (Peng et al., 2014; Sun et al., 2014; Boukir et al., 2019; Liu et al., 2019; Posoknistakul et al., 2020)  Intermolecular hydrogen-bonded H–O–H stretching  ѵ_as_CH_2_ and ѵ_s_CH_2_ in methylene and ѵ_as_CH_3_, ѵ_s_CH_3_ in methyl groups (Peng et al., 2014; Posoknistakul et al., 2020; Zhang et al., 2020)  ѵC=O in cyclic conjugated structure (quinone or p-quinone) (Boukir et al., 2019; Jiménez-López et al., 2020; Sun et al., 2021)  indicative of aromatic ring skeleton of lignin (Sun et al., 2021)  δ_s_O-H stretching vibration in glucuronic acid group (Sun et al., 2014)  C-O vibration in syringyl derivatives (Jiménez-López et al., 2020; Sun et al., 2021)  ѵC-O syringyl nuclei in lignin and hemicellulose (Wang et al., 2017; Boukir et al., 2019)  δ_as_C-O and δ_as_C–O–C asymmetric stretch vibration in hemicelluloses and dextran (Peng et al., 2014; Wang et al., 2017)  ѵC-O and ѵC-C or δC-OH in hemicelluloses (Peng et al., 2014; Sun et al., 2014; Liu et al., 2019)  indicative of arabinofuranosyl (Sun et al., 2014)  ѵC-O-C-glycosidic linkage of glucan and some monosaccharides (Wang et al., 2017; Boukir et al., 2019)  C-H out of plane bending of 1,2,4-tetrasubstituted aromatic in positions 2 and 6 of S units, and in all positions of H units (Wang et al., 2017; Boukir et al., 2019; Cao et al., 2021) |

**Table S2.** Assignments of main NMR signals (Wen et al., 2013; Huang et al., 2019; Fan et al., 2021; Huang et al., 2021).

| **Lable** | **δ_C_/δ_H_ (ppm)** | | **Assignments** |
| --- | --- | --- | --- |
|  | **Lignin extracted from BL** | **Alkaline lignin** |  |
| –OCH_3_ | 56.36/3.70 | 56.38/3.78 | C−H in methoxyls |
| A_γ_ | 59.8-60.5/  3.36-3.60 | 60.4-60.7/  3.41-3.63 | C_γ_−H_γ_ in β-O-4′ substructures (A) |
| C_γ_ | 63.73/3.87 | 63.41/3.64 | C_γ_−H_γ_ in phenylcoumaran substructures (C) |
| A_α_ | 72.3/4.83 | 71.77/4.79 | C_α_−H_α_ in β-O-4′ units (A) |
| A_β_ | - | 84.79/4.30 | C_β_−H_β_ in β-O-4′ units (A) |
| A_β(G/H)_ | 84.7/4.25 | - | C_β_−H_β_ in β-O-4′ substructures linked to G/H units (A) |
| A_β(S)_ | 86.74/3.97 | - | C_β_−H_β_ in β-O-4′ substructures linked to S units (A) |
| B_α_ | - | 85.73/4.63 | C_α_−H_α_ in β-β′ resinol substructures (B) |
| S_2,6_ | 104.41/6.69 |  | C_2,6_−H_2,6_ in syringyl units (S) |
| G_2_ | 111.5/6.96 | 112.21/6.91 | C_2_−H_2_ in guaiacyl units (G) |
| G_5_ | 115.15/6.68 | 116.02/6.78 | C_5_−H_5_ in guaiacyl units (G) |
| G_6_ | 119.67/6.77 | 119.43/6.77 | C_6_−H_6_ in guaiacyl units (G) |
| H_2,6_ | 128.40/7.18 | 128.40/6.97 | C_2,6_−H_2,6_ in H units (H) |
| PCA_2,6_ | 130.38/7.50 | - | C_2,6_−H_2,6_ in p-coumarate substructures (PCA) |
| FA | - | 128.78/7.22 | C−H in ferulate (FA) |
| FA_2_ | 111.71/7.27 | - | C_2_−H_2_ in ferulate (FA) |
| FA_6_ | 121.8/7.04 | - | C_6_−H_6_ in ferulate (FA) |
| FA_α_ | 144.62/7.48 | - | C_α_−H_α_ in ferulate (FA) |
| PCA_α_ | 144.62/7.48 | - | C_α_−H_α_ in p-coumarate substructures (PCA) |
| X_5_ | 62.3/3.46 | - | C_5_−H_5_ in β-D-xylopyranoside（X） |
| X_2_ | 73.23/3.04 | - | C_2_−H_2_ in β-D-xylopyranoside（X） |
| X_3_ | 74.50/3.26 | - | C_3_−H_3_ in β-D-xylopyranoside（X） |
| X_4_ | 75.90/3.51 | - | C_4_−H_4_ in β-D-xylopyranoside（X） |

**Table S3.** Tensile testing results of composite films.

| **Sample** | **Tensile strength (MPa)** | **Elongation at break (%)** |
| --- | --- | --- |
| PVA | 44.95±0.84 | 263.32±6.08 |
| PVA/AL-0.5 | 46.19±1.40 | 218.75±31.93 |
| PVA/AL-1.0 | 44.87±1.74 | 344.28±22.72 |
| PVA/AL-3.0 | 34.80±1.80 | 262.81±13.40 |
| PVA/AL-5.0 | 28.04±2.36 | 74.58±7.11 |
| PVA/AL-10.0 | 27.37±1.27 | 2.39±0.67 |
| PVA/BL-0.5 | 23.20±2.20 | 363.67±34.34 |
| PVA/BL-1.0 | 36.93±3.58 | 373.12±29.13 |
| PVA/BL-3.0 | 52.70±1.55 | 413.32±36.61 |
| PVA/BL-5.0 | 29.50±2.35 | 275.56±3.61 |
| PVA/BL-10.0 | 14.02±2.47 | 96.97±24.99 |

**Figure S1.** Alkalinities of various NaOH/water mixtures.

**Figure S2.** Water Absorption **(A)** and Swelling Properties **(B)** of various films.

**Reference**

Boukir, A., Fellak, S., and Doumenq, P. (2019). Structural characterization of Argania spinosa Moroccan wooden artifacts during natural degradation progress using infrared spectroscopy (ATR-FTIR) and X-Ray diffraction (XRD). *Heliyon* 5(9)**,** e02477. doi: 10.1016/j.heliyon.2019.e02477.

Cao, Q., Wu, Q., Dai, L., Li, C., Zhong, Y., Yu, F., et al. (2021). Size-controlled lignin nanoparticles for tuning the mechanical properties of poly(vinyl alcohol). *Industrial Crops and Products* 172. doi: 10.1016/j.indcrop.2021.114012.

Fan, Y.F., Li, L.H., Yang, G.X., Sun, Y.M., He, L.S., Wu, P.W., et al. (2021). Suppression Effect of Gamma-Valerolactone on the Mild Alkaline Pretreatment of Hybrid Pennisetum. *Acs Sustainable Chemistry & Engineering* 9(44)**,** 14846-14856. doi: 10.1021/acssuschemeng.1c04924.

Huang, C.X., Wang, X.C., Liang, C., Jiang, X., Yang, G., Xu, J., et al. (2019). A sustainable process for procuring biologically active fractions of high-purity xylooligosaccharides and water-soluble lignin from Moso bamboo prehydrolyzate. *Biotechnology for Biofuels* 12. doi: ARTN 189

10.1186/s13068-019-1527-3.

Huang, J.B., Guo, Q., Zhu, R.N., Liu, Y.Y., Xu, F., and Zhang, X.M. (2021). Facile fabrication of transparent lignin sphere/PVA nanocomposite films with excellent UV-shielding and high strength performance. *International Journal of Biological Macromolecules* 189**,** 635-640. doi: 10.1016/j.ijbiomac.2021.08.167.

Jiménez-López, L., Martín-Sampedro, R., Eugenio, M.E., Santos, J.I., Sixto, H., Cañellas, I., et al. (2020). Co-production of soluble sugars and lignin from short rotation white poplar and black locust crops. *Wood Science and Technology* 54(6)**,** 1617-1643. doi: 10.1007/s00226-020-01217-x.

Liu, X., Luan, S., and Li, W. (2019). Utilization of waste hemicelluloses lye for superabsorbent hydrogel synthesis. *Int J Biol Macromol* 132**,** 954-962. doi: 10.1016/j.ijbiomac.2019.04.041.

Peng, F., Guan, Y., Zhang, B., Bian, J., Ren, J.L., Yao, C.L., et al. (2014). Synthesis and properties of hemicelluloses-based semi-IPN hydrogels. *Int J Biol Macromol* 65**,** 564-572. doi: 10.1016/j.ijbiomac.2014.02.003.

Posoknistakul, P., Tangkrakul, C., Chaosuanphae, P., Deepentham, S., Techasawong, W., Phonphirunrot, N., et al. (2020). Fabrication and Characterization of Lignin Particles and Their Ultraviolet Protection Ability in PVA Composite Film. *ACS Omega* 5(33)**,** 20976-20982. doi: 10.1021/acsomega.0c02443.

Sun, S.-C., Sun, D., Li, H.-Y., Cao, X.-F., Sun, S.-N., and Wen, J.-L. (2021). Revealing the topochemical and structural changes of poplar lignin during a two-step hydrothermal pretreatment combined with alkali extraction. *Industrial Crops and Products* 168. doi: 10.1016/j.indcrop.2021.113588.

Sun, S.N., Cao, X.F., Li, H.Y., Xu, F., and Sun, R.C. (2014). Structural characterization of residual hemicelluloses from hydrothermal pretreated Eucalyptus fiber. *Int J Biol Macromol* 69**,** 158-164. doi: 10.1016/j.ijbiomac.2014.05.037.

Wang, F.-L., Li, S., Sun, Y.-X., Han, H.-Y., Zhang, B.-X., Hu, B.-Z., et al. (2017). Ionic liquids as efficient pretreatment solvents for lignocellulosic biomass. *RSC Adv.* 7(76)**,** 47990-47998. doi: 10.1039/c7ra08110c.

Wen, J.L., Xue, B.L., Xu, F., Sun, R.C., and Pinkert, A. (2013). Unmasking the structural features and property of lignin from bamboo. *Industrial Crops and Products* 42**,** 332-343. doi: 10.1016/j.indcrop.2012.05.041.

Zhang, X., Liu, W., Liu, W., and Qiu, X. (2020). High performance PVA/lignin nanocomposite films with excellent water vapor barrier and UV-shielding properties. *Int J Biol Macromol* 142**,** 551-558. doi: 10.1016/j.ijbiomac.2019.09.129.
